# P-373. The VREy Resistant Enterococcus: Retracing VRE Transmission and Contamination Events in Healthcare Settings

**DOI:** 10.1093/ofid/ofae631.574

**Published:** 2025-01-29

**Authors:** Tierney O’Sullivan, William Brazelton, Karim Khader, Molly Leecaster, Windy Tanner, Michael Rubin, Matthew H Samore, Lindsay T Keegan

**Affiliations:** University of Utah, Salt Lake City, Utah; University of Utah, Salt Lake City, Utah; University of Utah, Salt Lake City, Utah; University of Utah, Salt Lake City, Utah; University of Utah, Salt Lake City, Utah; University of Utah, Salt Lake City, Utah; University of Utah, Salt Lake City, Utah; University of Utah, Salt Lake City, Utah

## Abstract

**Background:**

Healthcare associated infections (HAIs), particularly those involving antibiotic resistant bacteria, are some of the most common, deadly, and preventable adverse events affecting patients in the clinical setting. Lowering HAI transmission rates would reduce the clinical burden to patients, economic burden to healthcare systems, and could help slow an alarming trend toward antimicrobial resistant organisms. However, determining definitive routes of transmission within healthcare facilities (HCFs) of HAIs is difficult and costly. Since HAIs are common in settings with little direct patient-patient contact, discerning the role of fomites in transmission pathways is critical, either via health care workers (HCWs) or from direct patient contact with contaminated surfaces.

**Methods:**

We present preliminary results of a model of who-infected-whom using densely sampled data from ICU units in two HCFs over 13 weeks. We combine daily microbiologic samples from three patient body sites, HCW hands, three zones within patient room environments, and shared environmental surfaces with epidemiologic data on HCW movement. Microbiologic samples were whole genome sequenced (WGS) for all distinct cultures. We developed a Bayesian transmission model that incorporates genomic and epidemiologic information to reconstruct probabilistic estimates of transmission routes to estimate who-infected-whom and rule out unlikely transmission or contamination events.

**Results:**

This poster focuses on results for the vancomycin-resistant enterococci (VRE) samples (N=44). We characterize the relationship between overlapping and non-overlapping patient stays, HCW hands, and environmental surfaces, and their roles in transmission and contamination within the two HCFs.

**Conclusion:**

Ultimately, these results provide further evidence on pathogen movement in HCFs and provide insights for developing and improving interventions to minimize transmission in HCFs.

**Disclosures:**

**All Authors**: No reported disclosures

